# Herpes Zoster Optic Neuritis: A Catastrophe of a Disease

**DOI:** 10.7759/cureus.60387

**Published:** 2024-05-15

**Authors:** Khairun Nisa Mohd Zaidan, Amirah Mohammad Razali, Mohamad Syafeeq Faeez Md Noh, Rafidah Md Saleh, Muhammad Mohd Isa

**Affiliations:** 1 Department of Ophthalmology, Hospital Sultan Abdul Aziz Shah, Universiti Putra Malaysia, Serdang, MYS; 2 Department of Ophthalmology, Faculty of Medicine & Health Sciences, Universiti Putra Malaysia, Serdang, MYS; 3 Department of Radiology, Hospital Sultan Abdul Aziz Shah, Universiti Putra Malaysia, Serdang, MYS; 4 Department of Radiology, Faculty of Medicine & Health Sciences, Universiti Putra Malaysia, Serdang, MYS

**Keywords:** neuro-ophtalmology, immunosupression, diabetes mellitus type 2, acute optic neuritis, herpes zoster ophthalmicus

## Abstract

Isolated herpes zoster optic neuritis is a rare sequelae of herpes zoster ophthalmicus (HZO). It can occur in the acute phase of HZO, or as post-herpetic complications. We report a case of a young patient with poorly controlled diabetes who developed herpes zoster optic neuritis one month after the initial skin manifestation despite completing a two-week course of oral acyclovir 800 mg five times a day. He complained of a five-day history of sudden onset, painless left eye blurring of vision. His vision over the left eye was no light perception with the presence of a left relative afferent pupillary defect. Fundus examination of the left eye revealed a swollen optic disc. Magnetic resonance imaging showed minimal fat streakiness over the left orbit. He was treated with one week of intravenous methylprednisolone 1 g/day, followed by a tapering dose of oral prednisolone (1 mg/kg/day) together with oral acyclovir 800 mg five times a day for another week. His visual acuity remained poor with a slight improvement in vision to hand motion.

## Introduction

Herpes zoster ophthalmicus (HZO) is a disease that occurs due to the reactivation of the herpes zoster infection in the dorsal root ganglion with retrograde migration involving the ophthalmic division of the trigeminal nerve [1]. Ocular complications occur in up to 78% of the cases with anterior segment structures mainly involved [1]. This includes adnexal involvement (58.8-73.8%), conjunctivitis (69.1%), episcleritis (11.9%), keratitis (31.4-59.5%), anterior uveitis (30.9-60.7%), and intraocular pressure elevation (23.5-42.86%) [1,2]. Posterior segment involvement is much less common such as posterior uveitis in 2% of cases [1]. Neuro-ophthalmic manifestations are also uncommon including oculomotor nerve palsy (3%), optic neuritis (1.9 to 4.8%), and orbital apex syndrome [1-3]. Most cases with poor visual outcomes are mainly due to keratitis, anterior and posterior uveitis, and optic neuritis [1]. Central nervous system involvement is a rare complication of HZO; a study in Denmark found that over seven years, only 5.5% out of 110 immunocompetent patients had neurological complications such as cranial nerve palsy, meningitis, and encephalitis [4].

Risk factors for herpes zoster infection can be innate causes such as race, sex, age, and family history [5]. The other risk factors are conditions causing immunosuppression, be it from diseases, infection, or medication. The most common infection is human immunodeficiency virus (HIV) infection and common chronic diseases include diabetes, asthma, chronic renal disease, and systemic lupus erythematosus (SLE) [5]. It has been reported that ocular manifestations occur in 87.1% of those above 45 years old and in those less than 45 years old, HIV seems to be the most common risk factor comprising 66.7% of patients with ocular manifestation [1]. Diabetes mellitus is among the commonly associated systemic diseases occurring in 20% of patients with ocular involvement [2].

We report a case of isolated optic neuritis secondary to HZO in a young patient with poorly controlled diabetes and poor visual outcome.

## Case presentation

**Figure 1:**
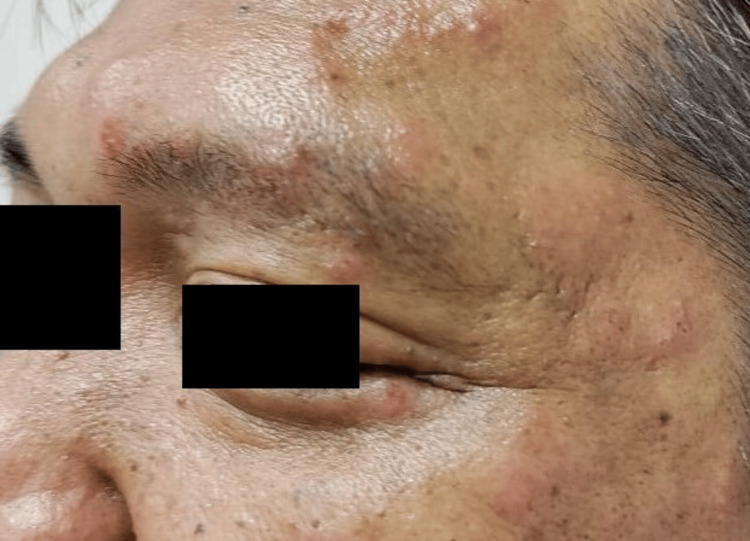
Left facial skin rashes involving the ophthalmic division of the trigeminal nerve.

A 45-year-old gentleman with underlying diabetes mellitus and hypertension, presented with a five-day history of sudden onset left eye painless blurring of vision. There was no associated redness, photophobia, floaters, eye pain, or pain on eye movement. Four weeks before the presentation, he developed a left-sided, vesicular facial rash over the ophthalmic division of the trigeminal nerve, not involving the tip of the nose (Figure 1). He was treated with oral acyclovir 800 mg five times a day for two weeks by the primary care doctor.

**Figure 2:**
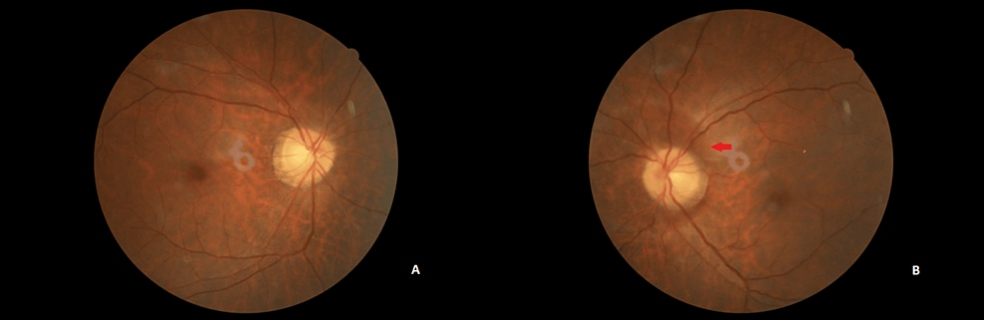
Fundus photo at presentation showing normal right eye (A) fundus findings and left eye (B) optic disc swelling with Paton’s line (red arrow).

On examination, his visual acuity was 6/6 over the right eye, and no light perception over the left eye. There was a left relative afferent pupillary defect. The anterior segment was unremarkable with intraocular pressure of 18 mmHg bilaterally. Fundus examination of the left eye revealed a swollen optic disc with the presence of Paton’s lines (Figure 2). The right eye fundus was normal. Extraocular movement was normal. Other neurological examinations were also normal.

Blood investigations showed a normal full blood count with no leucocytosis. The liver function test was also normal. The renal profile revealed slightly elevated creatinine of 125 umol/L with a normal urea level and a glomerular filtration rate of 59.5 ml/min. C-reactive protein was 11 mg/dL and the erythrocyte sedimentation rate was 5 mm/hour. He had poorly controlled diabetes with a fasting blood sugar level of 13.4 mmol/L with a glycated hemoglobin (HbA1c) level of 10.8%. Other blood investigations were also sent to rule out causes of atypical optic neuritis. The infective screening for toxoplasmosis, tuberculosis, syphilis, hepatitis B, hepatitis C, and HIV were negative. Connective tissue disease screening showed a low titer of 1:8 for antinuclear antibody and negative for both rheumatoid factor and anti-double strand antibody. He also tested negative for anti-myelin oligodendrocyte glycoprotein and anti-aquaporin 4 antibodies.

**Figure 3:**
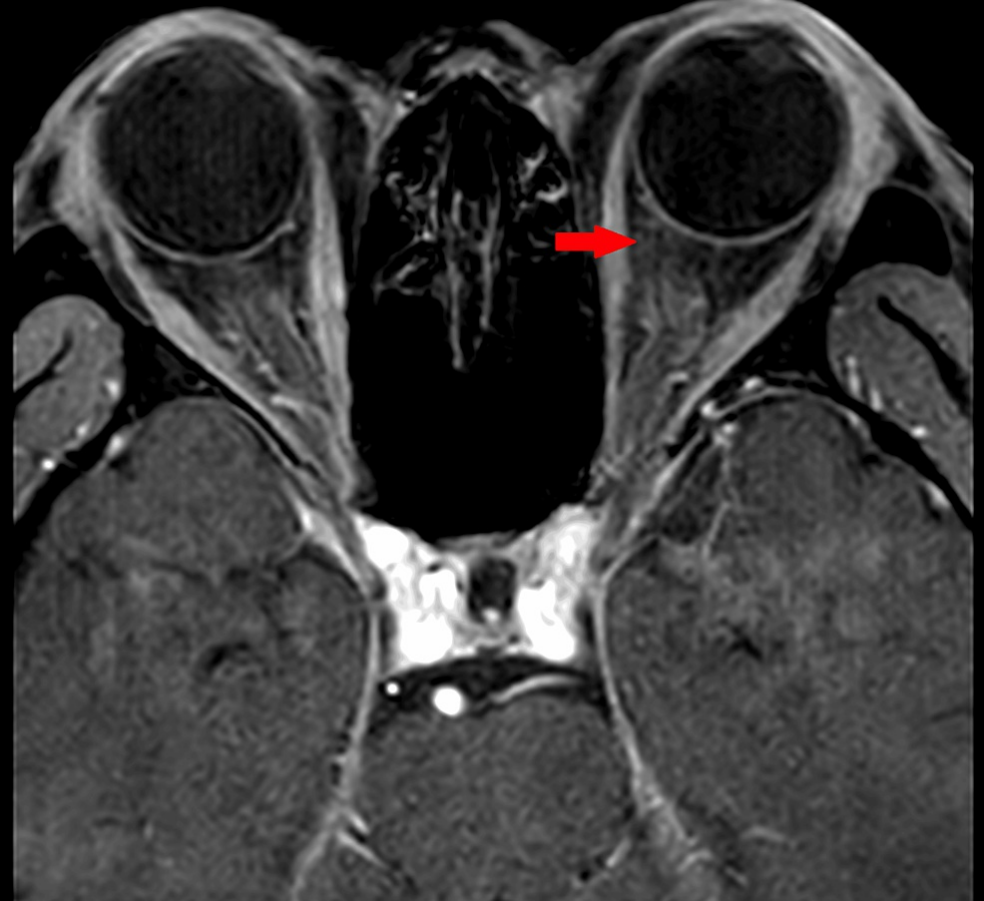
Magnetic resonance imaging orbit showing some fat streakiness over the left orbit (red arrow).

Magnetic resonance imaging of the brain was normal while the left orbit revealed only minimal perineural fat streakiness (Figure 3).

**Figure 4:**
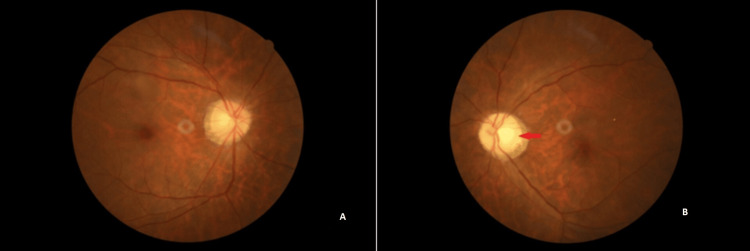
Fundus photo eight weeks after presentation showing a normal fundus on the right eye (A) and left eye (B) revealed a pale optic disc (red arrow).

A final diagnosis of herpes zoster-related optic neuritis was made. The patient was co-managed with the neuro-ophthalmologist and medical team. He was started on intravenous methylprednisolone 1 g daily for one week together with oral acyclovir 800 mg five times daily, followed by a tapering dose of oral prednisolone 1 mg/kg. His diabetic control was also optimized by the medical team and he was started on insulin therapy. Overall, there was only a slight improvement in vision from no light perception to hand motion after the commencement of treatment with a pale optic disc seen at eight weeks follow-up (Figure 4).

## Discussion

Herpes zoster optic neuritis (HZON) is one of the devastating complications of HZO, which may cause profound visual loss. It can develop acutely as early as six days or as post-herpetic complications after 30 to 45 days [6]. Kaufman et al. reported that 66% of HZON presented as retrobulbar optic neuritis whereas 33% manifested as anterior optic neuritis with optic disc swelling [6]. The majority of the cases have involvement of the ipsilateral optic nerve according to the laterality of facial rashes, while bilateral optic nerve involvement is relatively uncommon occurring in around 13.3% of cases [2]. This occurs more commonly in immunosuppressed patients but may also occur in immunocompetent patients [7,8]. It is postulated that the transsynaptic or intraneural spread of the virus in the optic nerve may be responsible for the bilateral involvement [7]. Most patients with HZON have concomitant ocular involvement. Isolated HZON such as that occurring in our patient is rare with only a few case reports available [6,9].

Normally, the varicella-zoster virus (VZV) is kept dormant in the dorsal root sensory ganglia by specific cell-mediated immunity. Reactivation of the VZV depends on various factors, but the greatest risk is associated with immunosuppression from HIV or malignancy [5]. Our patient did not have HIV or malignancy, but he did have poorly controlled diabetes leading to a relatively immunosuppressed state. Diabetes can worsen the immune system in various ways. It can affect both innate and adaptive immunity. Innate immunity is jeopardized when a state of hyperglycemia impairs the complement system, dendritic cells, macrophages, natural killer cells, natural killer T cells, and innate lymphoid cells through various mechanisms involving inflammatory mediators [10]. On the other hand, adaptive immunity is impaired by the impaired function of humoral immunity from the modified structure and function of immunoglobulin, leading to the formation of dysfunctional glycated antibodies that are unable to fight against the virus [10]. Other than that, T-cell functions are also damaged which includes both CD4+ and CD8+ T-cells [10]. This leads to diabetes patients being prone to virus infection and acquiring the more severe form compared to non-diabetics [10]. The exact pathogenesis of HZON is unknown but given the variable manifestations, it is most likely to be multifactorial. Gündüz and Ozdemir proposed three mechanisms: direct extension of the virus to the optic nerve, local extension via the meninges and brain tissue, and lastly from generalized ocular ischemia [7].

For neuroradiological characteristics in VZV reactivation involving the central nervous system, Maher et al. found that 44% of patients had enhancement of the optic nerve sheath consistent with optic perineuritis, 25% had optic neuritis, and 5.6% had papillitis [11]. Other radiological changes include cavernous sinus dural enhancement, single or multiple enhancing cranial nerves, encephalitis, small vessel vasculitis, and transverse myelitis [11]. Vanikieti et al. reported a case of isolated optic neuritis with concurrent abnormal trigeminal nucleus with magnetic resonance imaging showing enhancement and restricted diffusion of the optic nerve with linear hyperintense T2 of the ipsilateral spinal trigeminal nucleus and tract along the brainstem in a healthy patient with a final vision of counting fingers only [9]. The authors suggested that restricted diffusion of the optic nerve may be a predictor for poor visual recovery [9]. Overall, immunosuppressed patients are more likely to acquire significant central involvement such as encephalitis, vasculitis, or spinal or nerve root involvement [11]. Twenty-five percent of the immunocompetent patients with ocular and neuro-ophthalmic abnormalities did not have any magnetic resonance imaging changes [11].

There is no standard treatment for HZON. The mainstay of treatment involves the administration of systemic antiviral therapy, systemic steroids, or combination therapy. Both the dosage and the regime of the antiviral and the steroid therapy are variable based on the literature and are mainly tailored to the patient's clinical presentation. For herpes zoster treatment generally, antivirals are given within 72 hours of the rash onset with a dose of acyclovir of 800 mg five times daily, famciclovir of 500 mg three times daily, or valacyclovir of 1 gram three times daily for at least seven days. This helps reduce viral shedding, reduce new vesicle formation, shorten the duration of the infection, and reduce acute pain and postherpetic neuralgia [12]. Some patients despite being treated with the oral antiviral, continue to develop HZON and treatment has been variable with some patients receiving conversion to intravenous acyclovir 10 mg/kg every eight hours, continued with oral antiviral, or did not have further antiviral therapy after completion of the initial course, but instead was started on steroid treatment only [6]. For the steroid treatment, care has to be taken to ensure that the patient has no systemic contraindication before commencement. It is given as either intravenous methylprednisolone followed by oral prednisolone or given as oral prednisolone only with a variable tapering course of up to six weeks [6]. The role of an anti-inflammatory agent is essential in cases with intense inflammatory responses to the VZV antigen to prevent complications that may be associated with significant morbidity [11].

The prognosis of HZON seems variable depending on the extent of damage that occurred. Kaufman et al. in their series of HZON with the majority of patients having concurrent other HZO manifestations had a variable outcome with best corrected visual acuity of 6/6 to no light perception [6]. Prompt identification and treatment of HZON is crucial to improve outcomes [13,14]. Despite that, those with extensive optic nerve damage leading to optic atrophy or those with concurrent ischemic optic neuropathy usually have poorer visual prognosis and outcome [8,9,15]. Our patient was treated with oral acyclovir and systemic steroids, and despite that, extensive damage occurred leaving him with a pale optic disc and a poor vision of hand motion only. Hence, at-risk patients must be identified and given the VZV vaccine to reduce the incidence of infection and its associated complications [16]. Although there are reports of optic neuritis following vaccination, they are rare, and complete recovery has been reported with just the use of intravenous methylprednisolone [17]. 

## Conclusions

Isolated optic neuritis is a rare complication of herpes zoster ophthalmicus which may lead to profound vision loss. Treatment with antiviral therapy and systemic steroids may help improve the vision if given early in the majority of cases. Varicella zoster virus vaccination should be considered for at-risk populations to prevent such devastating complications.
